# Adhesive and non-adhesive internal hernia: clinical relevance and multi-detector CT images

**DOI:** 10.1038/s41598-019-48241-x

**Published:** 2019-09-06

**Authors:** Lei Dou, Huiyuan Yang, Chao Wang, Hao Tang, Dongjian Li

**Affiliations:** 1 Department of Surgery, Tongji Hospital, Tongji Medical college, Huazhong University of Science and Technology, Wuhan, China; 2 Department of Gerontology, Tongji Hospital, Tongji Medical college, Huazhong University of Science and Technology, Wuhan, China; 3 Department of Radiology, Tongji Hospital, Tongji Medical college, Huazhong University of Science and Technology, Wuhan, China

**Keywords:** Gastrointestinal diseases, Gastrointestinal diseases

## Abstract

Internal hernia (IH)-related surgical acute abdomen is not well understood because of the rarity of cases and underdiagnosis. This study was performed to further understand the clinicopathological features and multi-detector computed tomography (MDCT) findings of IH in cases confirmed by surgery. In all, 51 patients with a definite diagnosis of IH confirmed during surgical exploration from Feb. 2012 to Feb. 2018 in our hospital were included in this research. Medical records, including MDCT images and intra-operative findings, were collected retrospectively. In all, 39 and 12 cases were categorized as adhesive IH (76.5%) and non-adhesive IH (23.5%), respectively. Among the patients with adhesive IH, 73% had a history of abdominal or pelvic surgery. Additionally, the mesentery was the most common component of adhesive bands (64.1%). Congenital peritoneal abnormalities and gastrointestinal reconstruction were the main causes of non-adhesive IH.As a specific sign, the fat notch sign was much more common in adhesive IH than in non-adhesive IH (P = 0.023). Bowel wall thickening (P = 0.041), abnormal bowel wall enhancement (P = 0.006) and twisted bowels with the vessel swirl sign (P = 0.004) were indicators of bowel necrosis. Among all of the cases of IH, 34 (66.7%) were complicated by bowel necrosis, and 1 patient died. In conclusion, non-adhesive IH has different clinicopathological features and MDCT findings from those of adhesive IH. MDCT is a useful tool with high sensitivity for confirming IH and may help to guide the early treatment of IH.

## Introduction

Internal hernia (IH) is an acute or chronic protrusion of viscera through a primary or secondary hernia orifice formed by the mesentery, omentum, peritoneum or other organs. It accounts for 0.6 to 5.8% of small bowel obstruction cases. In previous studies, IH was usually categorized as primary (congenital) or secondary (acquired) IH. Idiopathic defects of the mesentery or omentum, paraduodenal fossae, and foramen of Winslow arethe main sites of the orifice of primary IH^1–3^. The orifice of secondary IH may be ascribed to adhesive band formation, gastrointestinal reconstruction, or trauma^4–6^. Some experts have also included diaphragmatic hernia in the IH category.

To date, there is no consensus onthe classification of IH. Some typical types of IH are determined by location, such as paraduodenal hernia, transmesenteric hernia and pelvic hernia. However, some studies have discussed specific types of IH according to aetiology, such as adhesive IH and IH after Roux-en-Y gastric bypass^7^. In fact, as adhesive IH represents a large proportion of cases, we classified adhesion IH as an independent category. Other cases of IH were classified as non-adhesive.

Adhesive IH can be further divided into primary and secondary groups according to the history of abdominal and/or pelvic surgery. Non-adhesive IH refers to cases of IH resulting from abnormalities of peritoneal structures. More specifically, non-adhesive IH can also be further divided into primary and secondary groups. In primary non-adhesive IH, a bowel segment protrudes into the normal peritoneal hiatus or a congenital defect in the peritoneal structure, such as in paraduodenal hernia and transmesenteric hernia. In secondary non-adhesive IH, the peritoneal structural defect is acquired, such as in IH after gastrointestinal reconstruction or trauma. Because diaphragmatic hernia and traumatic IH have distinctive clinical presentations, we do not discuss these two types of IH in this research.

As a rare cause of bowel obstruction, IH accounts for 0.6 to 5.8% of cases^8^. The majority of studies on this subject included a small number of cases or only focused on one specific type of IH^9^. However, in this work, which includes a large number of cases, we discuss the detailed clinical characteristics and MDCT findings of both adhesive and non-adhesive IH. We also further analyse the specific MDCT signs indicative of bowel necrosis. A comprehensive understanding of the multiple clinicopathological features and MDCT findings of IH would be helpful for guiding the diagnosis and early management of IH.

## Materials and Methods

### Patients

We retrospectively reviewed 51cases of IH confirmed by surgical exploration between Feb. 2012 and Feb. 2018. Medical records, especially the anatomical findings during surgery, were reviewed by 2 surgeons in consensus. MDCT images were reviewed retrospectively by two radiologists in consensus. The institutional review board of Tongji Hospital approved the study and did not require additional informed consent from the patients. All experiments were performed in accordance with the relevant guidelines and regulations for retrospective clinical studies. The names and admission numbers of the patients were replaced with codes for the protection of privacy.

Notably, 9 patients diagnosed with IH by clinical presentation and radiology, but no surgical exploration, we did not include these patients in this study.

### Multi-detector CT evaluation

All MDCT examinations were performed with a 64-MDCT scanner (Light Speed VCT SYS#CT99, GE Healthcare). A volume of 60–100 mL (1.5 mL/kg body weight) of nonionic iodinated contrast agent (iopromide 370 mg I/mL; Ultravist, Shanghai Bracco Sine Pharmaceutical) was injected, followed by 30 mL of saline solution at a rate of 3.5 mL/s with the use of a dual-head injector (Stellant D, Medrad). The section thickness was 5.0 mm, and the reconstruction interval was 0.625 mm. 2-dimensional reformatted images on sagittal planes add valuable information for optimal identification, such as the presence of hernia orifice and closed-loop bowels.

The MDCT features of IH were assessed for the following findings^10,11^: (1) Demonstration of bowel dislocation in IH: dislocated cluster of intestinal segments; crowding or convergence of mesenteric vessels. (2) Demonstration of obstruction or ischaemia: bowel wall thickening or abnormal enhancement; dilated bowels with abnormal free fluid;twisted bowels with the vessel swirl sign. (3) Demonstration of specific signs: the hernia orifice and closed intestinal loop; the fat notch sign.

Notably, 20 patients underwent urgent laparotomy, however, there were no MDCT images before surgery. We included these patients for the introduction of the clinical profile, but we did not include these 20 patients in the MDCT feature study.

### Definition

Adhesive bands are linear structures arising from one peritoneal structure or visceral organ to another, generating adhesive IH. Based on the history of abdominal (or pelvic) surgery, adhesive IH can be further divided into primary and secondary adhesive IH.

On MDCT, the fat notch sign indicates extra-luminal compression of the bowels by adhesive bands or a narrowed hernial orifice; the swirl sign indicates a twisted appearance formed by abnormal positioning of the mesenteric trunks^12^; and bowel wall thickening indicates that the thickness of the bowel wall is more than 3 mm in a dilated segment.

### Data collection and statistical description

The general demographics of the patients were collected, including sex, age, and body mass index (BMI). The preoperative data include the initial symptoms, the presence of acute bowel obstruction and the presence of peritonitis. The presence of bowel necrosis, components of the hernia orifice, and detailed anatomical structures of IH were also reviewed in the operative notes.

Summary statistics are expressed as the mean ± SEM, if appropriate. Patient characteristics are listed as the numbers of patients for items without specified units. The percentage was also listed for some parameters. Statistical comparisons between the 2 groups were performed using the chi-squared test (or Fisher exact test, where appropriate) for categorical data. All data analyses were performed using SPSS 22.0 statistical software. A two-sided P value < 0.05 was considered to indicate a significant difference.

## Results

### IH is a pleomorphic disease including several subtypes

As shown in Table 1, 39 (76.5%) and 12 (23.5%) patients were classified as having adhesive IH and non-adhesive IH, respectively, in this study. Of the 39 patients with adhesive IH, 24(61.5%) had a history of abdominal or (and) pelvic surgery. All patients had various degrees of abdominal pain. Other common clinical symptoms included abdominal distension and nausea/vomiting. Overall, 60–83%, 53.3–66.7%, and 28.6–54.2% of patients with different categories of IH suffered acute bowel obstruction, peritonitis and bowel necrosis. In total, 34 (66.7%) cases were complicated by bowel necrosis, and 1 patient died. The rate of bowel necrosis in the secondary adhesive IH (54.2%) group was higher than that in the other groups.

**Table 1:** Epidemiology and basic clinical features of IH in patients who undergone emergent surgery.

	Adhesive IH (n = 39)		Non-adhesive IH (n = 12)	
	Primary	Secondary	Primary	Secondary
No. of patients	15	24	7	5
Age(y)	52.7 ± 15.8	58.8 ± 18.6	49.0 ± 7.1	63.0 ± 4.7
Gender (M/F)	5/10	11/13	3/4	2/3
BMI(kg/m^2^)	23.1 ± 1.4	22.8 ± 0.8	22.6 ± 1.1	23.0 ± 1.2
Initial symptoms				
Abdominal pain	15	24	7	5
Abdominal distension	11	19	5	5
Nausia/vomiting	3	4	4	3
Acute bowel obstruction	11(73.3%)	20(83.3%)	5(71.4%)	3(60%)
Peritonitis	8(53.3%)	16(66.7%)	4(57.1%)	3(60%)
Bowel necrosis	6(40%)	13(54.2%)	2(28.6%)	2(40%)
Time from latest surgery(m)	—	60(2–276)	—	12(0.5–84)

Patient characters were listed as numbers of patients for items without specified units. Percentage was also listed in the table for some parameters. Time was listed as the median time and range.

### Hernia orifice may formed by various peritoneal structures and organs

Components of the hernia orifice in adhesive IH and the location of the hernia orifice in non-adhesive IH are shown in Table 2.

**Table 2:** Anatomical findings during surgery of IH patients.

**Components of adhesive bands in adhesive IH patients(n = 39)**	
Mesentery	25(64.1%)
Omentum	8(20.5%)
Mesocolon	4(10.3%)
Parietal peritoneum	13(33.3%)
Falciform	2(5.1%)
Appendix	5(12.8%)
Uterus or adnexa	4(10.3%)
**Location of hernia orifice in non-adhesive IH patients (n = 12)**	
Mesentery	2(16.7%)
Mesocolon	1(8.33%)
Omentum	1(8.33%)
Paraduodenal fossa	2(16.7%)
Broad ligament of the uterus	1(8.33%)
Retro-stoma space after Roux-en-Y gastric bypass	4(33.3%)
Retro-rectal space after Dixon operation	1(8.33%)

Patient characters were listed as numbers of patients for items without specified units. Percentage was also listed in the table.

Among the 39 patients with adhesive IH, components of the hernia orifice included the mesentery (64.1%), parietal peritoneum (33.3%), omentum (20.5%), appendix (12.8%), mesocolon (10.3%), uterus or adnexa (10.3%) and falciform (5.1%).

Among the12 patients with non-adhesive IH, the location of the hernia orifice included the retro-stoma space after Roux-en-Y gastric bypass, paraduodenal fossa, mesentery, mesocolon, omentum, broad ligament of the uterus and retro-rectal space after Dixon operation.

### Different subtypes of IH have similar patterns of MDCT signs except for the fat notch sign except for the fat notch sign

**Figure 1:**
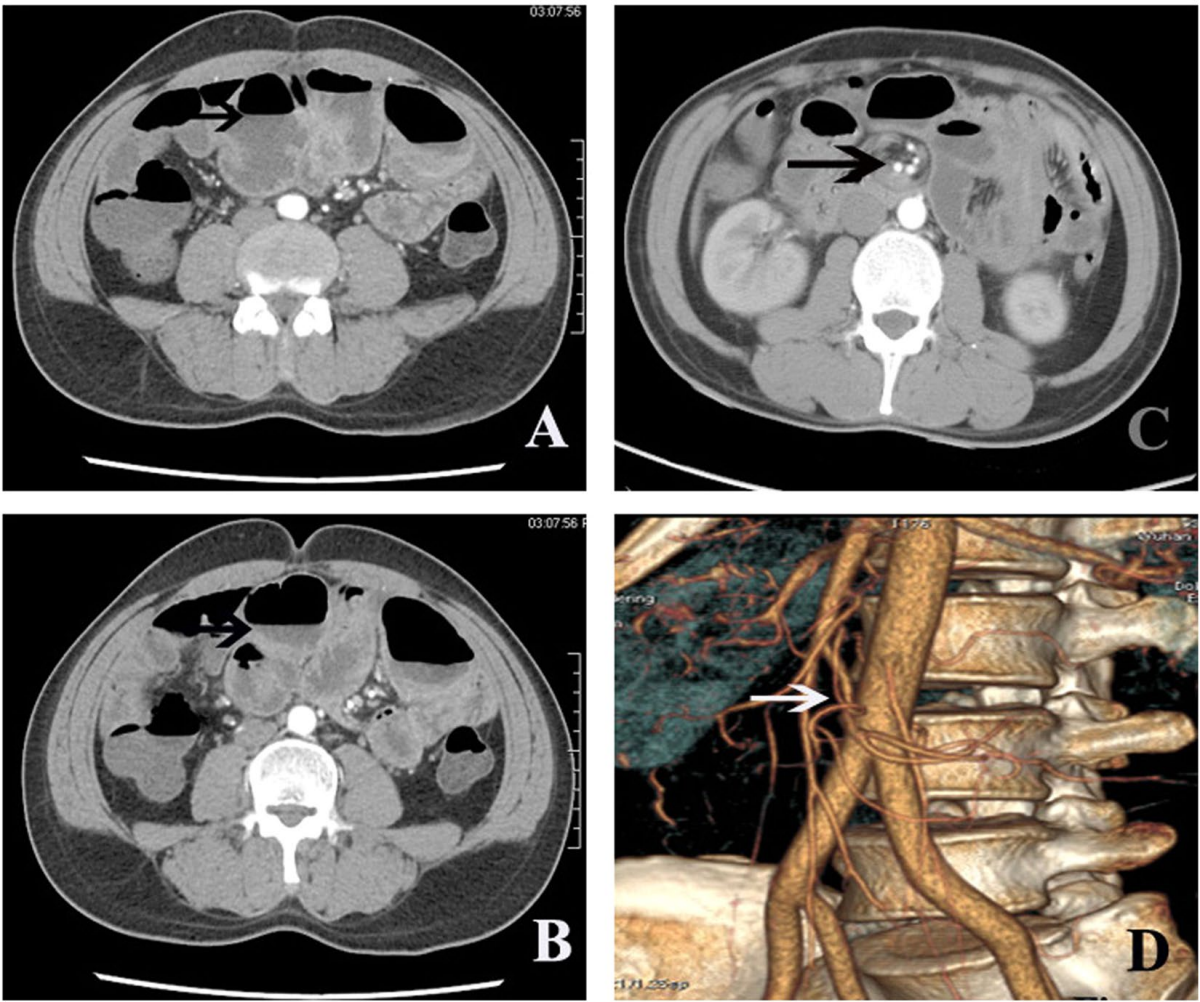
Some common non-specific MDCT signs in IH patients. (**A**,**B**) Axial MDCT images of a patient with adhesive IH showing the dilated bowel with abnormal free fluid (arrows). (**C**,**D**) Axial and vascular remodelling MDCT images of a patient with adhesive IH showing the vessel swirl sign (arrows).

The MDCT findings of the patients are also delineated in Table 3. Some features of MDCT signs in IH were also common to other conditions. For example, dilated bowels with abnormal free fluids, as shown in Fig. 1A,B, were also common features of bowel obstruction. Twisted bowels with the vessel swirl sign, as shown in Fig. 1C,D, may also be signs of bowel volvulus. Therefore, the identification of specific MDCT signs is crucial to the diagnosis of IH.

**Table 3:** Characteristic MDCT signs of IH patients.

	Adhesive IH (n = 21)	Non-adhesive IH (n = 10)	P
**Demonstration of bowels dislocation**			
Dislocated cluster of the intestinal segments	21(100%)	10(100%)	—
Crowding or convergence of mesenteric vessels	21(100%)	10(100%)	—
**Demonstration of bowel obstruction, ischemia or volvulus**			
Dilated bowels with abnormal free fluids	16(76.2%)	6(60.0%)	0.353
Bowel wall thickening	14(66.7%)	4(40.0%)	0.160
Abnormal enhancement of bowel	11(52.4%)	4(40.0%)	0.519
Twisted bowels with swirl sign of vessels	8(38.1%)	2(20.0%)	0.314
**Demonstration of specific signs**			
Hernia orifice	14(66.7%)	8(80.0%)	0.445
Fat notch sign	17(80.1%)	4(40.0%)	0.023

Patient characters were listed as numbers of patients for items without specified units. Percentage was also listed in the table. P value < 0.05 at two-sided was considered a significant difference.

**Figure 2:**
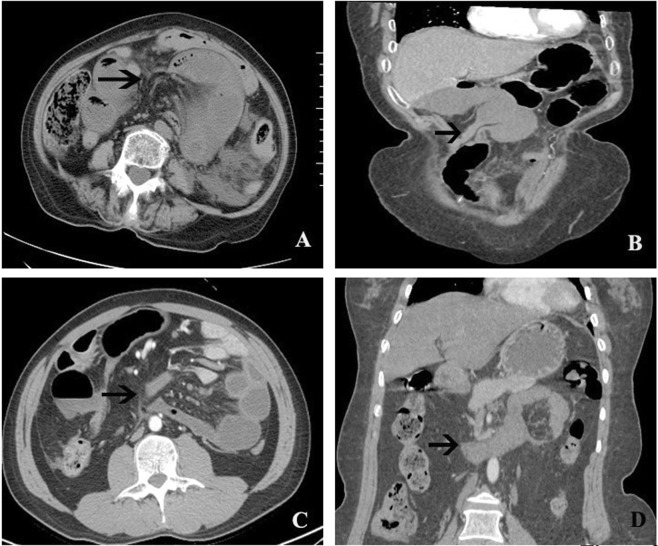
Representative MDCT images of patients with specific signs, including the hernia orifice and/or the fat notch sign. (**A**) The hernia orifice and the fat notch sign (arrow) in a patient with primary adhesive IH. The omentum and mesentery formed the adhesive band. (**B**) The hernia orifice and the fat notch sign (arrow) in a patient with secondary adhesive IH. The falciform ligament, omentum, and parietal peritoneum formed the adhesive band. (**C**) The hernia orifice and the fat notch sign (arrow) in a patient with secondary non-adhesive IH. The herniated bowel protruded via the mesenteric hiatus that formed after colectomy. (**D**) The hernia orifice (arrow) without the fat notch sign in a patient with primary non-adhesive IH, also known as paraduodenal hernia. The herniated bowel protruded into the Landzert fossa, which is an unusual congenital peritoneal defect behind the descending mesocolon.

The hernia orifice and the fat notch sign were recognized as specific MDCT signs for the diagnosis of IH. Representative patients are shown in Fig. 2. If either sign is identified by MDCT, a definite diagnosis of IH can be made by a radiologist. The hernia orifice was found in 14 (66.7%) and 8 (80%) patients in the adhesive and non-adhesive IH groups, respectively (P = 0.445). However, the fat notch sign was found in 17 (80.1%) and 4 (40%) patients in these 2 groups, showing a significant difference (P = 0.023).

### MDCT has higher diagnostic sensitivity than abdominal X-ray

Out of 51 patients with confirmed IH, 31 patients had pre-operative MDCT images. In these 31 patients, IH diagnoses were suspected in 25 patients before surgery. The diagnostic sensitivity of MDCT was 80.6%.

**Figure 3:**
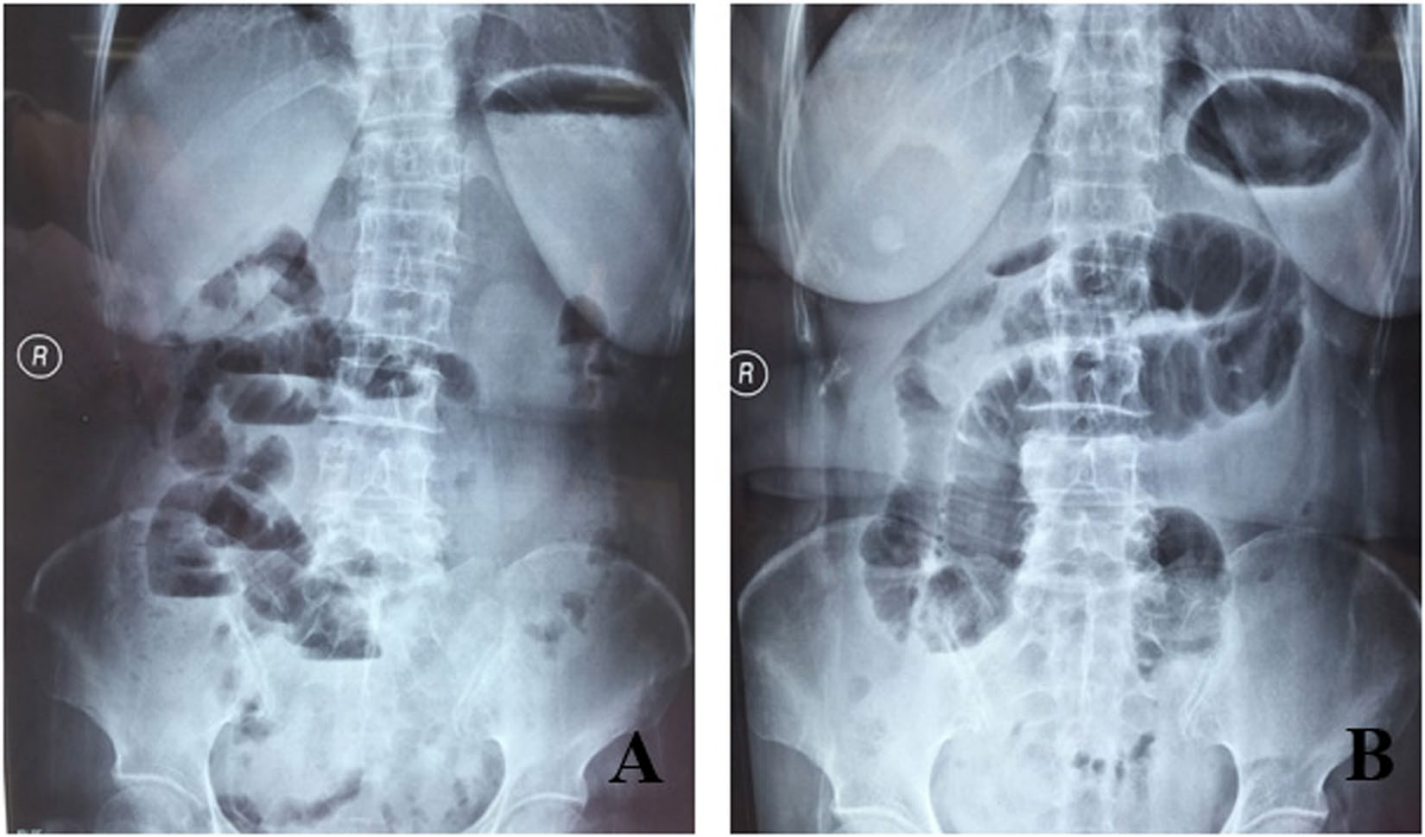
Two representative abdominal X-ray in 2 patients with intestinal. obstruction, but confirmed with IH by surgery. (**A**) one patient with multiple abdominal surgical history had abdominal pain and abdominal distension. Abdominal X-ray diagnosed with intestinal obstruction, surgery confirmed with IH diagnosis. (**B**) one patient had abdominal distension and vomiting at 4 days after radical resection of sigmoid colon cancer. Abdominal X-ray diagnosed with intestinal obstruction, surgery confirmed with IH diagnosis.

Out of 51 patients with confirmed IH, 49 patients had pre-operative abdominal X-ray images. In these 49 patients, IH diagnoses were suspected in only 14 patients on abdominal X-ray. The diagnostic sensitivity of abdominal X-ray was 28.6%. As shown in Fig. 3A,B, these 2 patients were diagnosed with intestinal obstruction before surgery, but IHs were confirmed by surgery.

### Indications of bowel necrosis complicating IH

Bowel necrosis is a life-threatening complication of IH that requires surgery for bowel resection. We further analysed differences in the MDCT signs between IH patients with and without bowel necrosis. As shown in Table 4, bowel wall thickening (P = 0.041), abnormal bowel enhancement (P = 0.006), and twisted bowels with the vessel swirl (P = 0.004) were considered characteristics of bowel necrosis in patients with IH. However, there were no differences in other MDCT signs between these 2 groups.

**Table 4:** Comparing of MDCT signs between IH with bowel necrosis and IH without bowel necrosis.

Multi-detector CT signs	IH with bowel necrosis	IH without bowel necrosis	p
	(n = 11)	(n = 20)	
**Demonstration of bowels dislocation**			
Dislocated cluster of the intestinal segments	11	20	—
Crowding or convergence of mesenteric vessels	11	20	—
**Demonstration of bowel obstruction, ischemia or volvulus**			
Dilated bowels with abnormal free fluids	7	15	0.505
Bowel wall thickening	10	8	0.041
Abnormal enhancement of bowel	10	11	0.006
Twisted bowels with swirl sign of vessels	8	4	0.004
**Demonstration of specific signs**			
Hernial orifice	8	14	0.873
Fat notch sign	6	15	0.244

Patient characters were listed as numbers of patients for items without specified units. P value < 0.05 at two-sided was considered a significant difference.

## Discussion

Because of the lack of reports of large groups of cases and standardized classification systems, there is still no consensus regarding the frequency of various types of IH. In this study, adhesive IH was the most common type of IH, accounting for 76.5% of cases. Of those patients, 61.5% had a history of abdominal or pelvic surgery. In one previous study on adhesive IH, 76.5% (26 out of 34) of patients had a history of surgery^4^. In the 7 patients with primary non-adhesive IH, left paraduodenal hernia occurred in 2 male patients. In one recent meta-analysis of 115 studies, including 159 patients, 69.8% and 30.2% of the patients suffered from either left or right paraduodenal hernia^13^.

Patients with secondary non-adhesive IH after gastrointestinal reconstruction have been investigated extensively. Roux-en-Y gastric bypass is the most frequent abdominal surgery that results in secondary non-adhesive IH^14^. Laparoscopic surgery may increase the occurrence of IH after surgery compared with traditional surgery^15^. The antecolic antegastric approach to Roux-en-Y gastric bypass is associated with fewer postoperative hernias than the retrocolic retrogastric approach. Meticulous attention to the closure of all mesenteric defects is crucial to decreasing the occurrence of this type of IH, especially in laparoscopic or robotic operations^16,17^. A prospective and randomized multicentre trial found that the application of fibrin glue may prevent the occurrence of IH after laparoscopic gastric bypass^18^. In this study, we confirmed 4 (33.3%) cases of non-adhesive IH due to Roux-en-Y gastric bypass surgery. Notably, 6 patients were also diagnosed with IH according to radiological tests after Roux-en-Y gastric bypass, but they were not included in this study because they did not receive surgical treatment.

Due to the lack of specific clinical symptoms, the early diagnosis of IH depends on radiological findings. MDCT has been proposed as a good diagnostic modality for the preoperative diagnosis of IH^19^. However, up to 20% of patients with IH may have negative CT findings for IH. Therefore, we should always keep in mind the possibility of IH even when MDCT does not show any specific evidence of IH. For different types of IH, the diagnostic efficacy of the same MDCT sign may be different. In this study, the fat notch sign was found in 81.0% of patients with adhesive IH and 40% of those with non-adhesive IH, showing a significant difference. Therefore, including the MDCT signs with the highest sensitivity and specificity in the decision tree is a focus for achieving the early diagnosis and proper treatment of IH. For example, superior mesenteric vein “beaking” and small bowel obstruction were identified as the most specific signs for the diagnosis of IH in patients who with a history of laparoscopic Roux-en-Y gastric bypass^11^.

Bowel necrosis is a common and life-threatening complication of IH. One patient in this study died because of the multiple organ dysfunction syndromes that result from severe bowel necrosis and peritonitis. Herniated bowels are prone to strangulation because of vascular compromise by compression of the hernia orifice or the presence of bowel volvulus. Bowel wall thickening and abnormal bowel enhancement were recognized as significant indicators of bowel necrosis, which isin accordance with previous reports^4,10^. In this study, twisted bowels with the vessel swirl sign, which is associated with bowel volvulus, was also a significant MDCT feature indicative of bowel necrosis. In total, 23 (45.1%) patients were definitively diagnosed with bowel necrosis and underwent concomitant bowel resection. For these patients with compromised bowels, early surgical intervention may be beneficial to avoid the development of bowel necrosis caused by IH.

Of course, our study has several limitations. First, this was a retrospective study and only reported IH patients confirmed by surgery. Some patients were not included in this study, such as those who were suspected to have IH but did not accept surgical intervention and those who were diagnosed incidentally during the operation. Therefore, the results should be considered with this information in mind. Next, although this study has one of the largest sample sizes and included some rare types of IH, it is still a single-centre study and could not include all types of IH. However, this study still provided a comparatively comprehensive understanding of IH.

## Conclusion

IH has a variety of anatomical and pathological manifestations. MDCT is a useful modality for the diagnosis and qualitative determination of IH. However, negative MDCT findings should not rule out a diagnosis of IH completely, especially in patients with a history of abdominal surgery. Prompt surgery in patients with MDCT findings of compromised bowels is necessary.
